# Correlating Agricultural Use of Organophosphates with Outdoor Air Concentrations: A Particular Concern for Children

**DOI:** 10.1289/ehp.7493

**Published:** 2005-05-13

**Authors:** Martha Harnly, Robert McLaughlin, Asa Bradman, Meredith Anderson, Robert Gunier

**Affiliations:** 1 California Department of Health Services, Oakland, California, USA; 2 University of California, Berkeley, California, USA; 3 Impact Assessment Inc., Oakland, California, USA

**Keywords:** agriculture, air, chlorpyrifos, chlorpyrifos oxon, diazinon, inhalation exposure, malathion, organophosphates, pesticides, volatilization

## Abstract

For the organophosphate pesticide chlorpyrifos, median inhalation noncancer, acute children’s exposures in agricultural communities are elevated above reference doses; for diazinon, similar exposures are nearly elevated. We used multivariate linear regression analysis to examine the temporal and spatial associations between agricultural use and measured air concentrations of chlorpyrifos, chlorpyrifos oxon, diazinon, and malathion. Agricultural use within a 3-mile radius on the monitoring day and use on the 2–4 prior days were significantly associated with air concentrations (*p* < 0.01) for all analytes except malathion; chlorpyrifos oxon showed the strongest association (*p* < 0.0001). In the final models, which included weather parameters, the proportion of variance (*r* ^2^, adjusted for the number of model variables) for all analytes ranged from 0.28 (*p* < 0.01) for malathion to 0.65 (*p* < 0.0001) for diazinon. Recent cellular, animal, and human evidence of toxicity, particularly in newborns, supports the public health concern indicated by initial risk estimates. Agricultural applications of organophosphates and their oxon products may have substantial volatization and off-field movement and are a probable source of exposures of public health concern.

Volatilization of pesticides from agricultural fields constitutes a large source of potential human exposure. The United States purchases a little more than 900 million pounds of active pesticidal ingredients annually for agricultural use (Donaldson et al. 2002), of which growers in California use approximately 16% [California Department of Pesticide Regulation (CDPR) 2005]. The amount volatized from agricultural fields can be considerable: for some pesticides, up to 90% of the application amount may volatize [reviewed by Bedos et al. (2002); Unsworth et al. (1999); Van den Berg et al. (1999)].

The pesticide air monitoring program of the California Environmental Protection Agency’s (Cal/EPA) Toxic Air Contaminant (TAC) Program is one of the few U.S. programs that monitor air in agricultural communities [Baker et al. 1996; U.S. Geological Survey (USGS) 1995]. In a health risk evaluation of the measured air concentrations, the broad-spectrum organophosphates chlorpyrifos and diazinon ranked highest in acute inhalation toxicity, after three fumigants with higher vapor pressures (e.g., methyl bromide). That evaluation derived the range (50th–95th percentiles) of inhalation risks, expressed as hazard quotients (HQs), the ratio of estimated intake to the reference dose (RfD). Estimated intakes were based on the distributions of air concentrations and inhalation rates for children (< 13 years of age) and adults. Organophosphate inhalation RfDs were based on the no- or lowest-observed-adverse-effect levels (NOAELs or LOAELs) in neurotoxicity animal studies of cholinesterase enzyme inhibition. For children’s acute and subchronic exposures, to derive the inhalation chlorpyrifos RfD (0.001 mg/kg/day), the NOAEL was divided by uncertainty factors totaling 1,000; to derive the diazinon RfD (0.00009 mg/kg/day), the LOAEL was divided by factors totaling 300. Risks were higher for children than for adults. For chlorpyrifos and diazinon, the HQs for the median of children’s acute exposures were 4.0 and 0.8, respectively. These results suggest a potential public health concern for residents and children of agricultural communities (Lee et al. 2002). The CDPR has placed both chlorpyrifos and diazinon on a high-priority list for risk characterization, which is a step toward listing pesticides as toxic air contaminants in California (CDPR and Patterson 2004).

Further specification of sources of ambient pesticide air concentrations is needed. For organophosphates, source or use patterns are complex. During the 1990s, use of chlorpyrifos and diazinon in residences or around public structures constituted up to 50 and 70%, respectively, of all agricultural and nonagricultural use [U.S. Environmental Protection Agency (EPA) 2000a, 2002].

Potential atmospheric dispersal and residence time of organophosphates after agricultural applications are not well understood and are driven by many interwoven factors, including application methods, tillage practices, irrigation techniques, temperature, sunlight, rainfall, and wind (Bedos et al. 2002; Racke 1993; Whang et al. 1993). Environmental fate estimates indicate that organophosphates volatize from surfaces and have short half-lives (i.e., a few days) on foliage, but in soil their half-lives vary widely (Table 1) [reviewed by Bradman et al. (1994); Miles et al. (1979); Racke (1993); Willis and McDowell (1987)]. Once in the air, the organophosphate phosphorothionates (a subclassification of organophosphates, including chlorpyrifos, diazinon, malathion, and others, e.g., azinphos-methyl, dimethoate, methidation, parathion, and phosmet) degrade within a matter of hours by reacting with photochemically produced hydroxy radicals (Hazardous Substances Data Bank 2002), degrading from thion (P = S) to oxon (P = O) compounds (Aston and Seiber 1997). The oxon degradation compounds are also formed in mammals, are more reactive, and are more potent inhibitors of acetylchlolinesterase than are the parent compounds (Chambers and Russell 1993; Huff et al. 1994; Poet et al. 2003). Spatially, pesticide air dispersion models estimate postapplication, off-field dispersal of ≤1 km (0.62 miles) after single applications of pesticides to specific agricultural fields (Raupach et al. 2001; Watanabe 2000). Yet deposition and some air measurements suggest that organophosphates may have an atmospheric dispersion of ≥100 km (62.1 miles) (Seiber et al. 1993; van Dijk and Guicherit 1999; Zabik and Seiber 1993).

In California, growers are required to report 100% of their agricultural pesticide use (CDPR 2000). For epidemiologic studies of chronic end points, such as childhood cancer and fetal outcomes, researchers have temporally and spatially aggregated the reported use as a broad indirect measure of many potential exposures (Bell et al. 2001; Reynolds et al. 2002). Yet no one has investigated whether there is a relationship between reported agricultural organophosphate use and human exposure by any pathway. For a health assessment of mothers and their children being conducted in California’s Salinas Valley [i.e., the CHAMACOS (Center for Health Analysis of Mothers and Children of Salinas) study (Eskenazi et al. 1999)], we plan to characterize media pathways of children’s organophosphate exposure from source to internal biologic dose. To address this aim, we used the chlorpyrifos, diazinon, and malathion air monitoring data collected by the Cal/EPA TAC program [California Air Resources Board (CARB) 1998a, 1998b, 1999]. Malathion is used widely in California but is lower in toxicity than other organophosphates because of mammalian metabolism (Ecobichon 1996). We characterized the associations between agricultural pesticide use and measured air concentrations at different temporal and spatial scales and examined the extent to which meteorologic conditions modify the relationships.

## Materials and Methods

### Air measurement data.

Air samples were collected by CARB for the CDPR and then analyzed by the Trace Analysis Laboratory at the University of California at Davis (UCD) Department of Environmental Toxicology, under contract with CARB. Their methods are described elsewhere (CARB 1998a, 1998b, 1999). In summary, during application season CARB placed monitors on the roofs of four accessible public buildings in populated high-pesticide-use areas. The areas monitored for chlorpyrifos and diazinon (Tulare and Fresno Counties, respectively) are in the southeastern portion of California’s Central Valley. Malathion monitoring occurred in Imperial County, an irrigated desert area. The distances between monitoring stations averaged 9.0, 9.1, and 13.8 miles for chlorpyrifos, diazinon, and malathion, respectively. At each monitoring location, the distance to nearby fields where target applications may occur was noted and varied between close (e.g., across the street) and 1 mile away.

Chlorpyrifos monitoring was conducted in the late spring of 1996, diazinon monitoring in the winter of 1998, and malathion monitoring in the early spring of 1998. CARB collected samples over 3-week (diazinon, malathion) and 4-week (chlorpyrifos) periods based on the use patterns for the respective pesticides. Generally, 24-hr samples were collected, using lipophilic polystyrene resin (XAD-4) beginning between 0800 hr and 1000 hr, Monday through Friday, with a flow rate of 15 L/min for chlorpyrifos and a lower flow rate (3–4 L/min) for diazinon and malathion (CARB 1998a, 1998b, 1999). Quality control included field and trip spikes, trip blanks, and replicate samples. Analytical measurements included spiking the resin tube with labeled analyte before extraction. In their analysis for chlorpyrifos and chlorpyrifos oxon, UCD used a gas chromatograph with a flame photometric detector and a filter for phosphorus detection; quantification was confirmed with an electrolytic conductivity detector and/or a mass selective detector operated in selective ion monitoring mode (CARB 1998a). For diazinon and malathion, UCD used a gas chromatograph with a DB-17 capillary column and a quadrapole mass spectrometer operated in selected ion mode (CARB 1998b, 1999). The method quantitation limits (QL) per sample were 68 ng for chlorpyrifos, 113 ng for chlorpyrifos oxon, 44.5 ng for diazinon, 17.3 ng for malathion, and 34.0 ng for maloxon. All blanks were less than the QL. Acceptable recoveries, ranging from 77 to 125%, were reported for all spiked analytes. Similar to other pesticide air measurements in California (Lee et al. 2002), results were not corrected for recovery. The relative differences of co-located samples, where amounts in both samples were above the QL, had mean relative differences of 12% for chlorpyrifos (16 pairs), 11% for chlorpyrifos oxon (16 pairs), 4% for diazinon (8 pairs), and 15% for malathion (11 pairs; CARB 1998a, 1998b, 1999).

### Meteorologic data.

We extracted meteorologic data (temperature, wind speed, wind direction, and rainfall) from the California Weather Database, maintained by the University of California Statewide Integrated Pest Management Program (UC-SIPMS 2003). We selected the meteorologic station closest to each air monitoring location and for which appropriate weather data had been collected for the monitoring period. Two chlorpyrifos and two diazinon monitoring locations matched to meteorologic stations nearby (within 3 miles), whereas the two other chlorpyrifos and two other diazinon monitoring locations matched to stations farther away (9 and 15 miles). All four malathion monitoring locations matched to stations 5–9 miles away.

### Pesticide data.

CDPR performs a number of validity checks—for example, duplicate records and valid pesticide and geographic identifiers—and maintains a public-use data set of the mandatory agricultural pesticide application reporting known as Pesticide Use Reporting. Growers report their use by date, application method (aerial vs. ground), and section of the Public Land Survey System (PLSS); a PLSS section is approximately 1 mi^2^. Nonagricultural applications (i.e., at residences, schools, and golf courses) are not reported by PLSS sections and could not be included in our analysis (CDPR 2000, 2005). To evaluate local impacts, we retrieved pesticide-use data for an approximate 49-mi^2^ area surrounding each of the 12 monitoring sites. The small percentage of records (< 1%) flagged by CDPR as probable errors or outliers, that is, inordinately high applications, were deleted (CDPR 2000). We divided each 49-mi^2^ area into two smaller areas: an inner circle encompassing the PLSS sections within a 1.5-mile radius of the monitoring site and a doughnut, or outer circle, encompassing the PLSS sections between 1.5 and 3 miles from the monitoring site. We summed pesticide use within the inner and outer circles on each sample collection day (day *i*). To evaluate how earlier use affected the air concentration on day *i*, we also summed use separately in the inner and outer circles on each of the 3 days before the monitoring day (day *i* – 1, day *i* – 2, and day *i* – 3). A 4-day temporal selection is consistent with the range of midpoints (3 and 5 days) of reported half-lives on foliage (Table 1). Except for chlorpyrifos models, which had a larger sample size, and where we included day *i* – 4 in the inner circle, we included neither earlier use nor use in a broader area because the limited number of samples collected would not support a larger number of variables in a multivariate analysis (Harrell 2001).

### Statistical analyses.

Using SAS (version 8; SAS Institute Inc., Cary, NC), we generated stepwise multiple linear regression models. For collected air samples below the QL, we entered half that limit. Air concentrations followed a lognormal distribution and were log-transformed for regression analyses. To examine the impact of pesticide use, we developed two series of multiple regression models, entering use variables stepwise. The first series of models examined use applied per square mile in the inner and outer circles combined (pounds per square mile in PLSS sections within a 3-mile radius). We generated a univariate model of pounds applied in the combined inner and outer circles on day *i*. Then, to determine whether use on the day before the sample collection day explained additional variance in air concentrations, we generated a separate model with the two variables, use on day *i* and use on day *i* – 1. Continuing to build separate regression models with additional variables, we repeated this stepwise process for each of the days before the monitoring day (days *i* – 2 and *i* – 3). After adding each variable, we tested for the overall fit of the model with the *F*-test (where *F* is the ratio of the regression mean square to the deviation mean square). To determine the significance of an added variable (i.e., whether the coefficient for the added variable was nonzero), we performed a log-likelihood ratio test. We did not remove variables based on these *p*-values because *p*-value–based variable selection may violate statistical estimation principles (Harrell 2001). We did review the multivariate model: When the proportion of variance (*r* ^2^), adjusted for the number of variables in our analysis, decreased after addition of a pesticide use variable, or when the parameter estimates on any use variable in the model became negative, we removed the variable from subsequent stepwise models. To evaluate whether pesticide use in the inner and outer circles had independent impacts, we similarly generated a second series of models but entered use in the inner and outer circles as two variables.

Using the model series for each analyte that generated the highest *r* ^2^, we continued the described stepwise multiple regression modeling procedure, adding variables that might modify the relationship between use and air concentrations including: percent pounds applied upwind of monitor (percentage of pounds applied in the upwind PLSS section on day *i*, as determined by the average wind direction, of the total pounds applied in both circles), percent pounds applied aerially in the PLSS sections within both circles on day *i*, monitor location, maximum daily temperature, average daily wind speed, and average daily rainfall. To code each monitor location uniquely and parsimoniously, we created two dichotomous indicator variables: For chlorpyrifos and diazinon, this was north versus south and east versus west of the centroid of the four monitors; for malathion, this was north versus south and, because the two northern monitors were also both western monitors, the monitors closest to the centroid of the four monitors versus those farther away. Although for chlorpyrifos and diazinon, which were monitored at the base of the Sierra Nevada Mountains, the directional coding may capture the influence of diurnal wind patterns (e.g., nighttime cooling and movement of foothill air), we primarily considered these monitor variables as proxy indicator variables that could control for several unmeasured factors (i.e., structural or landscape pesticide use near the monitor, terrain, or other unknown factors related to the monitor location). Residuals of final models were confirmed to follow a normal distribution.

## Results

**Figure 1:**
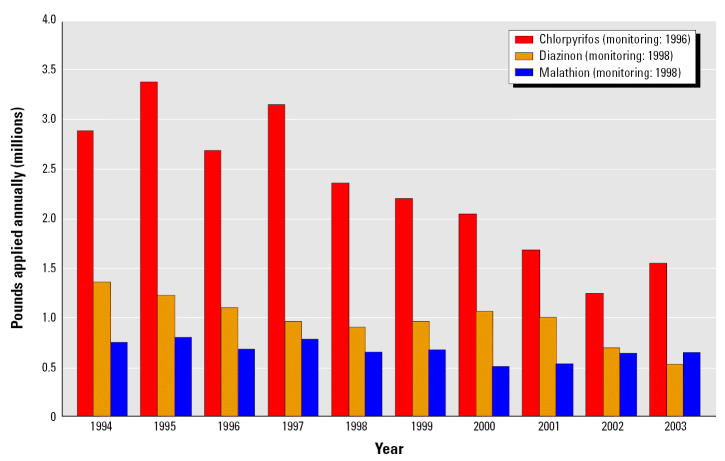
Agricultural use of organophosphates in California by year.

**Figure 2:**
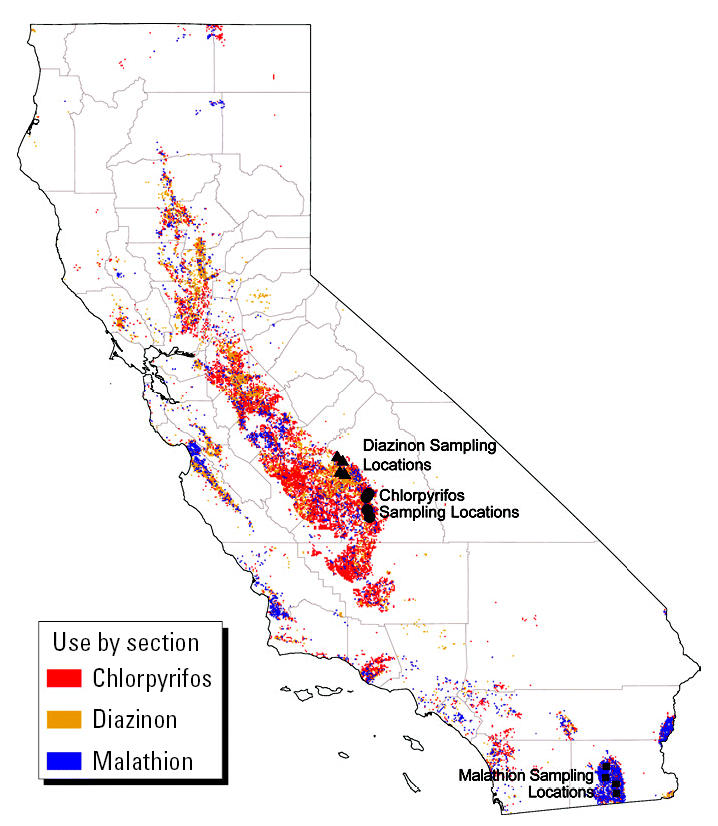
Map of organophosphate (chlorpyrifos, diazinon, malathion) use and locations of monitors, in California.

Pesticide use in California, during the years that air monitoring occurred, was generally similar to use in other years (Figure 1). Sampling locations were in areas of heaviest pesticide use (Figure 2). Of the three pesticides we examined, chlorpyrifos had the highest use statewide and the widest geographic distribution, followed by diazinon and then malathion.

Table 2 shows the average use, air concentrations, and weather variables in each monitoring area. Chlorpyrifos had the highest use and the highest air concentrations. Chlorpyrifos samples were collected during the warm, late spring, and diazinon samples during the foggy, rainy winter dormant season. Malathion monitoring also occurred during the winter, but the desert conditions of the monitoring location were warm and dry.

For the oxon breakdown products, chlorpyrifos oxon was detected in 83% of samples. Diazoxon was not measured. Maloxon was measured but was above the QL in only 46% of samples—an insufficient number for statistical analysis.

All applications of chlorpyrifos and diazinon were ground applied. Malathion was applied both on the ground and by air. Because each of the three pesticides was applied predominantly to one crop (Table 2) in the monitored areas, we could not include a target-crop variable in our analysis.

Tables 3 and 4 show the proportion of variance explained (adjusted *r* ^2^) for our multiple regression models. For both chlorpyrifos and chlorpyrifos oxon, the associations between measured air concentrations and agricultural pesticide use are stronger for our second series of use models, where uses in the inner and outer circles are separate variables. Chlorpyrifos oxon air concentrations are significantly associated with use in the inner and outer circles on the monitoring day (day *i*) and adding use on the prior day (day *i* – 1) improves the adjusted *r* ^2^. Adding use on day *i* – 2, day *i* – 3, and day *i* – 4 in the inner circle but not the outer circle further improves the adjusted *r* ^2^. For chlorpyrifos, there is a similar relationship, although the overall fit of the model is less and not significant until most of the use variables are in the model. The better overall fit for the second series of models compared with the first suggests independent impacts of use in the inner compared with the outer circles.

Adding the location of the monitor to the chlorpyrifos model (Table 4) improves the adjusted *r*^2^. Adding weather parameters (i.e., wind speed), slightly improves the *r*^2^ for both chlorpyrifos and chlorpyrifos oxon. The significance of the log-likelihood tests that the coefficient of each added variable is nonzero ranges from 0.002 for the addition of use within inner circle day *i* – 3 to the chlorpyrifos oxon model (an increase in *r*^2^ from 0.31 to 0.39), to 0.17 for the addition of use within the outer circle, day *i* – 2, to the chlorpyrifos oxon model (an increase in *r*^2^ from 0.24 to 0.25). For the final models, the adjusted *r*^2^ is 0.30 for chlorpyrifos (*p* < 0.01 for overall fit) and 0.43 for chlorpyrifos oxon (*p* < 0.0001 for overall fit).

For diazinon and malathion, the associations between measured air concentrations and pesticide use are stronger for our first series of models, where use in the two use areas is combined as one variable (Table 3). For diazinon, the adjusted *r*^2^ improves both when use for day *i* – 1 is added and when use for day *i* – 2 is added. Notably, the addition of monitor location and daily temperature (positively correlated with air concentrations) increases the *r*^2^ for the diazinon model to 0.38 (for overall fit, *p* < 0.0001), and when either wind speed or rainfall (both negatively correlated with air concentrations and highly correlated with each other) is added, the final *r*^2^ increases to 0.65 (for overall fit, *p* < 0.0001) (Table 4). In the diazinon final model, the tests that the coefficient of each added variable is nonzero reveal statistical significance (*p* = 0.05 to < 0.0001) for each added variable.

For malathion, the adjusted *r*^2^ improves when pesticide use on day *i* – 1 is added and is similar to that seen for chlorpyrifos, although the overall fit of the multivariate model is not significant because of the smaller number of samples collected (Table 3). Adding use on day *i* – 2 and day *i* – 3 marginally improves the *r*^2^. Adding the percent pounds applied upwind, the monitor location, and weather parameters to the model (Table 4) each improves the adjusted *r*^2^. The *p*-values of the tests that the coefficient of each added variable are nonzero, ranging from 0.03 for the addition of wind speed to 0.24 for the addition of pesticide use, day *i* – 2. The adjusted *r*^2^ for the final model is 0.28 (for overall fit, *p* < 0.01).

## Discussion

This study demonstrates associations between regional agricultural application quantities of organophosphates and measured air concentrations. National pesticide-use maps suggest that areas of high agricultural use for the three organophosphates we studied are found in many states (USGS 1997). Our results also suggest analyte priorities and potential spatial and temporal parameters for inhalation exposures to organophosphates.

More specifically, measured air concentrations of chlorpyrifos oxon showed a stronger association with reported agricultural use than did chlorpyrifos. This stronger relationship is consistent with the longer estimated air half-life of chlorpyrifos oxon (11 hr) compared with chlorpyrifos (4 hr) (Aston and Seiber 1997). The 24-hr sample collection period studied here allows for the photochemical reaction to occur.

The detectable impact of organophosphate use on community air concentrations may be brief, on the order of days: Inclusion of day *i* – 3 in our models often failed to improve the adjusted *r*^2^ over the association shown by inclusion of days *i* through *i* – 2 (Table 3). This finding is consistent with the lower range of the reported half-lives on foliage (Table 1). Other studies are also supportive: In a Canadian agricultural area, chlorpyrifos air levels dropped notably in the week after applications (Rawn and Muir 1999); on each of the 2 days after the day of urban aerial applications of malathion, air levels of the pesticide were approximately halved (Brown et al. 1993). Nonetheless, an initial period of high volatilization may be followed by a longer period of slower volatilization (Brown et al. 1993; Glotfelty et al. 1990b; Whang et al. 1993) that could not be studied here because of the limited sampling scope.

For all analytes, the adjusted *r*^2^ improves by including pesticide use in square-mile sections out to a 3-mile radius (5,000 m), whether as a single combined-use variable or independently as two separate variables. This distance is large compared with that studied after single applications on specific agricultural fields: Researchers have generally measured or estimated air concentrations at 10 feet to one-half mile (800 m) from the field (Raupach et al. 2001; Watanabe 2000; Woodrow et al. 1997). There is, however, considerable potential for the atmospheric persistence and long-range movement of vapor and droplets of semivolatile pesticides through vaporization of larger to smaller droplets, vertical air mixing resulting in increased droplet and vapor height, and deposition of droplets with subsequent re-entry into the atmosphere (Van den Berg et al. 1999; van Dijk and Guicherit 1999). In studies of organophosphate ambient air in the Sierra Nevada mountains and fog water of the Salinas Valley, oxon and thion parent organophosphates were detected 9–14 miles (15–22 km) from application sites, with oxons in higher concentration than the thion parent products (Aston and Seiber 1997; Schomburg et al. 1991).

Inclusion of monitor location, entered as proxy indicator variables controlling for unmeasured factors related to the location, improved the adjusted *r*^2^ for most of the pesticide models (Table 4). Several unmeasured factors are possible, including locational differences in application or other farming practices, topography that could affect diurnal wind flow, and pesticides applied to buildings near the monitors. Inclusion of weather parameters, most notably wind speed, also improved the models in the predicted direction, particularly for diazinon. Because diazinon monitoring was conducted in winter, during windy, rainy, and heavy fog conditions, inclusion of wind speed may account for washout or dilution of background air concentrations, making the impact of agricultural use more apparent. The water solubility of diazinon has been noted (Glotfelty et al. 1990b), and in our analysis, rainfall was highly correlated with wind speed; inclusion of either factor equally improved the *r*^2^.

There is considerable unexplained variance in our models. However, there are many inherent limitations of the data. The number of samples and locations was limited. Meteorologic data were not specific to the air monitoring location, and the amounts in colocated samples also varied some (on average, 4–15%), suggesting analytical variability in air measurements. The fraction of analyte bound to airborne particulates was not measured, although this has been shown for organophosphates to be < 5% of the mass (Glotfelty et al. 1990a).

Underreporting of pesticide use is another potential source of uncertainty. A systematic evaluation of underreporting (i.e., a field survey of applications to validate use reporting) has never been conducted. However, growers are required by law to report their pesticide use, and local agricultural commissioners may inspect use reporting when they conduct audits of pesticide applicator records or when investigating potential misapplication of pesticides (California Office of Administrative Law 2001). Another limitation is that we could not examine whether applications closer to the monitor (i.e., < 1 mile) had a greater impact, and we could not include in our models data on pesticide application methods (e.g., orchard blast vs. ground application) or farming practices such as tilling and irrigation. Given the limited sampling size and scope, as well as the many factors we could not evaluate, our models did explain a considerable fraction of the variability of measured pesticide air concentrations. Our results suggest that agricultural applications of organophosphates are a source of exposure, that significant impacts may be brief (on the order of days), that the spatial dispersion may be greater than that currently studied in single-pesticide-application air studies, and that oxon degradation compounds may be equally, if not more, important contributors to air concentrations than the parent thion products.

Others have noted the need for improved pesticide ambient air monitoring (Carey and Kutz 1985; USGS 1995). Improved air monitoring and enhanced pesticide-use reporting, including enhanced geographic resolution and farming practices, could not only validate estimated exposure zones and duration but also provide valuable regulatory tools for estimating the amount of pesticide use reduction necessary to achieve a desired air level.

Organophosphates have been the focus of a variety of regulatory efforts to reduce exposures. Based on estimated dietary risks, chlorpyrifos was recently withdrawn for use on tomato and apple crops (U.S. EPA 2000a). In California, educational outreach to growers has reduced organophosphate use on orchards (Epstein et al. 2001). Nonetheless, consideration of the air exposure pathway in the recent U.S. EPA chlorpyrifos reregistration risk assessment was waived based on estimated photodegradation; the more toxic oxon metabolite was not considered (U.S. EPA 2000b). In contrast, the recent U.S. EPA risk evaluation of diazinon suggested that greater consideration of the air pathway was needed (U.S. EPA 2002). In our study, chlorpyrifos had greater use and higher air concentrations than diazinon, and those concentrations represented a higher health risk than those for diazinon (Lee et al. 2002). These results suggest that the U.S. EPA environmental fate and pesticide-use estimates should be reexamined.

Whether initial estimates of elevated inhalation risks for chlorpyrifos and diazinon exposures to children (Lee et al. 2002) are adequately protective is uncertain. Although intra- and interspecies 10-fold uncertainty factors were used, the true range of mammalian response is unknown (Hattis et al. 1996). Children and adults may alone differ by several orders of magnitude in their susceptibility (Eskenazi et al. 1999; Faustman et al. 2000). Recent comparison of RfDs to risks quantified by examining the entire range of the animal dose–response curve (i.e., benchmark doses) demonstrates that RfDs generally underestimate health concern and do not represent negligibly small risks (Castorina and Woodruff 2003). Greater evidence of the vulnerability of embryos, fetuses, neonates, and adolescents to organophosphates has also recently emerged. In murine embryos, cell death has been induced at the chlorpyrifos RfD for drinking water, 0.003 mg/kg/day (Greenlee et al. 2004). Additional organophosphate neurodevelopmental toxicity mechanisms include gliogenesis, axonogenesis, and synaptogenesis (Qiao et al. 2002). Chlorpyrifos oxon also binds directly to some (i.e., muscarinic) cholinergic receptors (Huff et al. 1994). In humans, among 314 newborns in New York City, low levels (1–4 pg/g) of chlorpyrifos and diazinon in umbilical cord plasma were inversely associated with birth weight and length (Whyatt et al. 2004). Although these outcomes were not correlated with maternal personal air levels of chlorpyrifos and diazinon, the oxon metabolites were not measured in air. Further, the personal air levels were significantly correlated with the cord blood levels and were, for chlorpyrifos, lower (chlorpyrifos mean = 15 ng/m^3^) than that studied here (Table 2) (Whyatt et al. 2004).

We have studied only a few organophosphates. In 2003, in addition to the 2.7 million pounds of chlorpyrifos, diazinon, and malathion applied to California agriculture, 1.2 million lb of other organophosphorothionates, and a total of 7.9 million lb of cholinesterase inhibitors (i.e., all organophosphates and carbamates), were applied on California agriculture (CDPR 2005). Our findings suggest that reducing agricultural organophosphate use could reduce community air concentrations and lower inhalation risks to children and residents living in and around agricultural communities.

## Figures and Tables

**Table 1:** Environmental fate parameters.

Parameter	Chlorpyrifos	Diazinon	Malathion
Vapor pressure (mm Hg at 25°C)	2.0 × 10^–5^^a^	9.0 × 10^–5^^a^	1.8 × 10^–4^^b^
Water solubility (mg/L)	1.4 at 25°C^a^	60 at 20°C^a^	145 at 25°C^a^
Half-life			
Air as vapor (hr)	4^c^	4^c^	5^c^
Foliage (days)	1–9^d^	1–5^e^	< 1–9^f^
Soil (days)	2–1,575^d^	3–87^g^	1–6^f^

a Data from Tomlin 1997.

b Data from Daubert and Danner 1989.

c Data from Hazardous Stubstances Data Bank 2002.

d Reviewed by Racke (1993).

e Reviewed by Willis and McDowell (1987).

f Reviewed by Bradman et al. (1994);

g Data from Miles et al. 1979.

**Table 2:** Monitoring conditions and median air concentrations.

Parameter	Chlorpyrifos	Diazinon	Malathion
County of monitoring	Tulare	Fresno	Imperial
Major crop	Citrus	Almond	Alfalfa
Monitoring period	28 May–29 June 1996	12 Jan–2 Feb 1998	23 Feb–12 Mar 1998
No. of monitoring days	20	12	12
No. of monitoring locations	4	4	4
Percent samples above QL^a^	84	60	83
Median air concentration^b^	33 ng/m^3^	17 ng/m^3^	9.9 ng/m^3^
Mean lb/mi^2^ per day			
Within 1.5 mi	9.9	1.2	4.1
Within 1.6–3 mi	7.7	1.4	3.2
Mean (range) applications/day, within 3 mi	3.2 (0–16)	3.9 (0–27)	1.5 (0–11)
Average maximum daily temperature (°F)	89	58	73
Average daily wind speed (mi/hr)	3.8	4.1	4.9
Mean daily inches of rainfall	0	0.12	0.004

a Percentage of chlorpyrifos oxon samples above QL = 83%.

b Median air concentration for chlorpyrifos oxon = 22 ng/m^3^.

**Table 3:** Stepwise multiple regression models, adjusted *r*^2^,^a^ of air concentrations and pesticide use (lb/mi^2^).

Variables added sequentially^b^	Chlorpyrifos	Chlorpyrifos oxon	Diazinon	Malathion
Model series 1^c^				
Use within 3 miles: day *i*	0.07	0.13*	0.12*	0.08
Use within 3 miles: day *i* – 1	0.08	0.21#	0.17*	0.14
Use within 3 miles: day *i* – 2	0.09	0.24#	0.23**^d^	0.14
Use within 3 miles: day *i* – 3	Removed^e^	Removed^e^	Removed^e^	0.15^d^
Model series 2^f^				
Use within 1.5 miles: day *i*	0.05	0.16#	0.01	0.05
Use within 1.6–3 miles: day *i*	0.07	0.19#	0.05	0.07
Use within 1.5 miles: day *i* – 1	0.09	0.24#	0.06	Removed^e^
Use within 1.6–3 miles: day *i* – 1	Removed^e^	0.25#	0.1	0.13
Use within 1.5 miles: day *i* – 2	0.13*	0.31#	0.12	Removed^e^
Use within 1.6–3 miles: day *i* – 2	Removed^e^	Removed^e^	0.13	0.16
Use within 1.5 miles: day *i* – 3	0.18*	0.39#	0.14	Removed^e^
Use within 1.6–3 miles: day *i* – 3	Removed^e^	Removed^e^	Removed^e^	Removed^e^
Use within 1.5 miles: day *i* – 4	0.21^d^	0.41^#d^	NA	NA

NA, not applicable (i.e., analysis was not conducted).

a Adjusted for the number of variables in the model; statistical significance is a test of the overall significance of the multivariate model.

b Each row within a model series represents a regression model with the named variable (the added variable) and the variables named in the rows above it included. Day i is the sample collection day. Day i – 1, i – 2, i – 3, and i – 4 represent each of the 4 days before the sample collection day.

c For model series 1, pesticide use in PLSS sections within a 3-mi radius of monitor was entered as one variable.

d Pesticide use variables in this model selected for subsequent analysis.

e The adjusted *r*^2^ decreased compared with previous model or the use parameter coefficients were negative; the variable was eliminated from subsequent models.

f For model series 2, use in PLSS sections within a 1.5- and a 1.5- to 3-mi radius of monitor were entered as two separate variables.

* p < 0.01;

** p < 0.001;

# p < 0.0001.

**Table 4:** Addition of weather/locational variables to stepwise multiple regression models, pesticide use variables included: adjusted *r*^2^ for multivariate model.^a^

Additional variables added sequentially^b^	Chlorpyrifos^c^	Chlorpyrifos oxon^c^	Diazinon^d^	Malathion^d^
Percent pounds applied upwind	Removed^e^	Removed^e^	Removed^e^	0.18
Percent pounds aerially applied	NA	NA	NA	Removed^e^
Location of monitor	0.29#	Removed^e^	0.29**	0.22
Temperature (maximum daily)	Removed^e^	Removed^e^	0.38#	Removed^e^
Wind speed (average daily)	0.30#^f^	0.43#^f^	0.65#^f^	0.28*^f^
Rainfall (average daily)	NA	NA	Removed^e^	Removed^e^

NA, not applicable (i.e., there were either no applications applied aerially or there was no rainfall).

a Adjusted for the number of variables in the model; statistical significance is a test of the overall significance of the multivariate model.

b Each row within a model series represents a regression model with the pesticide use variables, the variable named in the row (the added variable) and the variables named in the rows above it included.

c Model series 2 (pesticide use out to 3-mi radius as two separate variables) generated improved adjusted *r*^2^ compared with model series 1 (pesticide use out to 3-mi radius as one variable), and the additional variables are stepwise added to model series 2.

d Model series 1 (use out to 3-mi radius as one variable) generated improved adjusted r 2, compared with model series 2, and additional variables are added stepwise to model series 1.

e Adjusted *r*^2^ decreased or remained the same, or any use parameter coefficients were negative; the variable was eliminated from subsequent models.

f Final model.

* p < 0.01;

** p < 0.001;

# p < 0.0001.
